# SIRT1 suppresses the migration and invasion of gastric cancer by regulating ARHGAP5 expression

**DOI:** 10.1038/s41419-018-1033-8

**Published:** 2018-09-24

**Authors:** Guoying Dong, Bo Wang, Yifei An, Juan Li, Xin Wang, Jihui Jia, Qing Yang

**Affiliations:** 1 Institute of Pathogen Biology, School of Basic Medical Sciences, Shandong University, Jinan, 250012 China; 2 Department of Traditional Medicine, Qilu Hospital, Shandong University, Jinan, 250012 China; 3 Department of Clinical Laboratory, The People’s Hospital of Huaiyin, Jinan, 250021 China

## Abstract

Gastric cancer (GC) ranks among the top five malignant tumors worldwide by the incidence and mortality rate. However, the mechanisms underlying its progression are poorly understood. In this study, we investigated the role of SIRT1, a class III deacetylase, in the invasion and metastasis of GC. Here, we found that knockdown of SIRT1 promoted GC cell migration and invasion in vitro and metastasis in vivo. Forced expression of SIRT1 in GC cells had the opposite effects. Then, we used mRNA microarray to identify the target genes that are regulated by SIRT1 and found that *ARHGAP5* was downregulated by SIRT1. The results of the mRNA microarray were confirmed in several GC cell lines. Furthermore, SIRT1 inhibited the expression of *ARHGAP5* by physically associating with transcription factor c-JUN and deacetylating and inhibiting the transcriptional activity of c-JUN. Then the expression dynamics and clinical significance of ARHGAP5 were analyzed using clinical samples and database. The expression of ARHGAP5 was increased in GC, and positively correlated with tumor size, tumor infiltration, lymph node metastasis, and clinical stage. And multivariate analyses indicated that ARHGAP5 served as an independent prognostic marker of GC. In addition, the biological effects of ARHGAP5 in SIRT1-mediated inhibition of GC migration and invasion were investigated using both in vitro and in vivo models. Silencing of *ARHGAP5* considerably inhibited the migration and invasion of GC, and ARHGAP5 was found to be involved in the SIRT1-mediated inhibition of GC migration and invasion. Our results indicate that SIRT1 suppresses migration and invasion of GC by downregulating *ARHGAP5* through an interaction with c-JUN, and these phenomena represent a novel mechanism of the antitumor action of SIRT1.

## Introduction

Gastric cancer (GC) ranks among the top five malignant tumors worldwide by the incidence and mortality rate^1^. In 2012, it was estimated that 951,600 new GC cases and 723,100 GC-related deaths occurred around the world^2^. The leading cause of death from GC is invasion and metastasis of tumor cells. This is because once the tumor reaches the advanced or metastatic stage, the present therapeutic strategies are largely ineffective^3^. Thus, it is urgently necessary to gain a better understanding of the molecular pathogenesis of GC metastasis to improve patients’ outcomes. The invasion and metastasis of a malignant tumor are complicated processes in which many genetic and epigenetic events are involved. Because of the dynamic nature of epigenetic changes, they are thought to play an important role in determining a metastatic phenotype^4^.

Sirtuin 1 (SIRT1), the founding member of the sirtuin family, was discovered as mammalian homolog of silent information regulator 2 (Sir2) in *Saccharomyces cerevisiae*, which was the first evolutionarily conserved gene to be demonstrated to act as a regulator of longevity in yeast^5,6^. SIRT1 possesses a NAD^+^-dependent deacetylase activity, and its substrates include not only histones but also nonhistone proteins^7–9^. By regulating different targets, SIRT1 has shown exciting connections between protein deacetylation and biological or pathological processes including energy metabolism, autophagy, inflammation, and cancer^10–14^. Our previous study has revealed an inhibitory role of SIRT1 in the development of GC via NF-κB-cyclin D1 signaling^15^. In addition to proliferation, SIRT1 participates in the invasion and metastasis processes of human malignant tumors. By deacetylating SMAD4, SIRT1 represses the effect of TGF-β signaling on MMP7 (a SMAD4 target gene) and consequently cell migration and tumor metastasis in breast cancer and oral squamous cell carcinoma^16,17^. In lung cancer and ovarian cancer, SUMO E3 ligase PIASy suppresses SIRT1 expression by reducing SP1 occupancy of the promoter of SIRT1 and thus promotes metastasis^18,19^. It is also reported that SIRT1 promotes proteasomal degradation of oncogenic transcription factor FOXM1 and thereby reverses the nutrient sensor O-linked-β-*N*-acetylglucosamine (O-GlcNAc) transferase (OGT)-mediated invasion and metastasis of breast cancer^14^. By present, the effect of SIRT1 on the invasion and metastasis of GC and the pathway involved is largely unknown.

In this study, we explored the role and the target genes of SIRT1 in the migration and invasion of GC. We found that SIRT1 suppressed the migration and invasion of GC in vitro and in vivo. Moreover, we validate that SIRT1 exerts these inhibitory effects by targeting *ARHGAP5* via physical interacting with and inhibiting the transcriptional activity of c-JUN. Therefore, our findings indicate that the SIRT1–c-JUN–ARHGAP5 axis may represent a novel mechanism underlying the progression and metastasis of GC.

## Materials and methods

### Cell culture and siRNAs

Human GC cell lines AGS, BGC-823, HGC-27, MGC-803, and SGC-7901 (Cell Resource Center, Institute of Biochemistry and Cell Biology at the Chinese Academy of Sciences, Shanghai, China) were cultured in F12 (AGS) or RPMI 1640 (BGC-823, HGC-27, MGC-803, and SGC-7901) containing 10% fetal bovine serum (FBS), 100 U/ml penicillin, and 100 µg/ml streptomycin. The cell bank routinely performs cell line authentication by short tandem repeat profiling, and all the cell lines were passaged in our lab for no more than 6 months after receipt. Lentiviral vectors containing SIRT1 cDNA, SIRT1 short hairpin (sh)RNA, ARHGAP5 shRNA, or their controls were constructed by GenePharma (Shanghai, China). Stably lentivirus-infected GC cells were obtained as described before^15^ and were cultured in complete medium supplemented with 1 µg/ml puromycin (Acros Organics, Belgium). Chemically modified small interference RNAs (siRNAs) targeting *c-JUN*, *RELA*, or *ARHGAP5* and control siRNA were bought from GenePharma. The sequences of the above siRNAs are listed in Supplementary Table 1. Lipofectamine 2000 (Invitrogen, Carlsbad, CA, USA) was used to transfect siRNAs.

### Wound healing assay

Cells were seeded in six-well plates and subjected to the experiment when they reached 80–90% confluence. Then, 20 µl pipette tips were used to scratch across the center of the wells. The plates were washed with phosphate-buffered saline three times before addition of fresh medium. The cell migration was examined and photographed at the initiation time point and at 16 and 24 h after the wounding. Three random visual fields were analyzed. The migration ratio was calculated from the ratio of a migratory distance to the initial distance. The assays were performed in triplicate and repeated three times.

### Tanswell assay

This assay was carried out in Transwell chambers (Costar, New York, NY, USA). Cells resuspended in medium containing 1% FBS were seeded into the upper chambers not coated (for migration assay) or coated with Matrigel (BD Biosciences, Franklin Lakes, NJ, USA) (for invasion assay). The lower chambers were filled with medium containing 20% FBS. After incubation for indicated periods, cells remaining in the chamber were removed, and cells on the lower surface of the membrane were fixed, stained with 0.05% crystal violet, and photographed. Five random visual fields were manually counted at ×200 magnification for each chamber, and three independent experiments were conducted for each group.

### mRNA microarray profiling

Stably lentivirus-infected BGC-823 cells were analyzed by a whole human genome oligo microarray. Sample labeling and array hybridization were performed according to the Agilent One-Color Microarray-Based Gene Expression Analysis protocol (Agilent Technology). The hybridized arrays were washed, fixed, and scanned on the Agilent DNA Microarray Scanner (part number G2505C).

### RNA extraction and quantitative PCR (qPCR)

Total RNA was extracted with TRIzol reagent (Invitrogen) and converted into cDNA as described^15^. The qPCR was performed for the following genes: *c-JUN, RELA, ARHGAP5, Bcl-xl, MMP7, c-FOS*, and *β-actin*. The primer sequences are listed in Supplementary Table 1. Calculation of the target mRNA levels was normalized to *β-actin* relative to the control by the 2^−ΔΔCt^ method. Experiments were conducted in triplicate and repeated three times.

### Western blot

Total cellular protein was isolated with RIPA Lysing Buffer (Beyotime, Jiangsu, China). The protein concentrations were measured with a BCA Protein Assay Kit (Pierce, Rockford, IL, USA). The membrane was probed with specific primary antibodies against SIRT1, ARHGAP5 (Abcam, Cambridge, MA, USA), and c-JUN (Cell Signaling, Danvers, MA, USA) and then with a horseradish peroxidase-conjugated anti-mouse or anti-rabbit IgG antibody. The protein bands were developed using the ECL system (Millipore, Boston, MA, USA). β-Actin (Cell Signaling) or GAPDH (Proteintech, Rosemont, IL, USA) served as the loading control.

### Luciferase assay

Luciferase reporter plasmids containing the promoter sequence of *ARHGAP5*, assumed c-JUN or RELA-binding sites deleted promoter sequences of *ARHGAP5* were constructed by Bioasia (Jinan, China). Cells were seeded in 24-well plates and transfected with the appropriate reporter plasmid via the X-tremeGENE HP Transfection Reagent (Roche Applied Science, Basel, Switzerland). Then 48 h after transfection, the cells were lysed, and firefly and renilla luciferase activities were measured with the Dual-Luciferase Reporter Assay Kit (Promega, Madison, WI, USA). Experiments were conducted in triplicate and repeated three times.

### Co-immunoprecipitation (co-IP) assay

AGS cells and stably lentivirus-infected AGS cells were harvested and lysed with NP40 lysis buffer (Beyotime). The proteins were precipitated with antibodies against c-JUN (Cell Signaling), SIRT1 (Abcam), or IgG (Cell Signaling). Next, protein G PLUS-Agarose beads (Santa Cruz Biotechnology, Santa Cruz, CA, USA) were added to the protein antibody mixture. The precipitated materials were collected, boiled, and analyzed by western blot as described above. The anti-acetylated-lysine antibody was purchased from Cell Signaling.

### Chromatin immunoprecipitation

AGS cells and stably lentivirus-infected AGS cells were crosslinked with 1% (v/v) formaldehyde-containing medium for 10 min at 37 °C, followed by sonication to make soluble chromatin. Chromatin immunoprecipitation (ChIP) assays were performed using the SimpleChIP^®^ Enzymatic Chromatin IP Kit (Cell Signaling) with anti-c-JUN (Cell Signaling), anti-SIRT1 (Millipore), or IgG (as a negative control). The purified DNA served as the template to amplify the *ARHGAP5* promoter. The primer sequences are listed in Supplementary Table 1. The amount of DNA precipitated by a specific antibody was compared with the corresponding input DNA.

### Mouse model

To set up a lung metastasis model, 5 × 10^5^ stably lentivirus-infected BGC cells of each group were injected into the tail vein of six-week-old male nude mice (Charles River, Beijing, China). Four weeks later, the nude mice were killed, and their lungs were dissected, weighed, fixed, and embedded for evaluation of metastasis. The average weight of lungs was calculated and compared among the groups. The lung specimens were stained with hematoxylin and eosin (HE). Nude mice without any treatment were regarded as normal. The animal protocol was approved by the Animal Ethics Committee, School of Basic Medical Sciences, Shandong University (No. 201402039).

### Tissue array and immunohistochemistry (IHC)

Commercial tissue microarray (catalog no. HStm-Ade180Sur-04) were purchased from Shanghai Outdo Biotech Company (Shanghai, China). This tissue microarray contained 180 tissue spots including 90 pairs of GC tissues and the corresponding adjacent non-cancerous mucosa. The clinicopathological and survival data of the patients were provided by the manufacturer. The informed consent signed by all the patients was kept by the manufacturer. This study was approved by the Ethics Committee, School of Basic Medical Sciences, Shandong University (No. 201401037). IHC staining was performed as previously described^15^. Specific primary antibody against ARHGAP5 (Abcam) was used for IHC. The intensity of ARHGAP5 stain was scored as follows: 0, no staining; 1, light brown; 2, medium brown; and 3, dark brown. The proportion of positively ARHGAP5 stained cells (0–100%) was recorded. The overall score of ARHGAP5 expression was calculated by multiplying the staining intensity by the positive percentages. Samples with an IHC score >200 were regarded as high expression, while those with an IHC score ≤200 were low expression.

### Statistical analysis

Comparisons between different groups were made by one-way ANOVA with Tukey’s post-hoc test or Student’s *t*-test. The relationships between ARHGAP5 expression and clinicopathological characters of GC patients were evaluated with *χ*^2^ test. Overall survival comparison between the high ARHGAP5 group and the low ARHGAP5 group was conducted by log-rank (Mantel–Cox) test in the Kaplan–Meier plots. The effects of the clinicopathological variables and ARHGAP5 levels on the patients’ survival were determined by the univariate and multivariate Cox proportional hazard regression model. Statistical analyses were performed using Statistical Package for Social Sciences, version 22.0 (SPSS Inc., Chicago, IL, USA). Data with *p*-values <0.05 were considered statistically significant.

## Results

### SIRT1 inhibits migration and invasion of GC cells in vitro

**Fig. 1:**
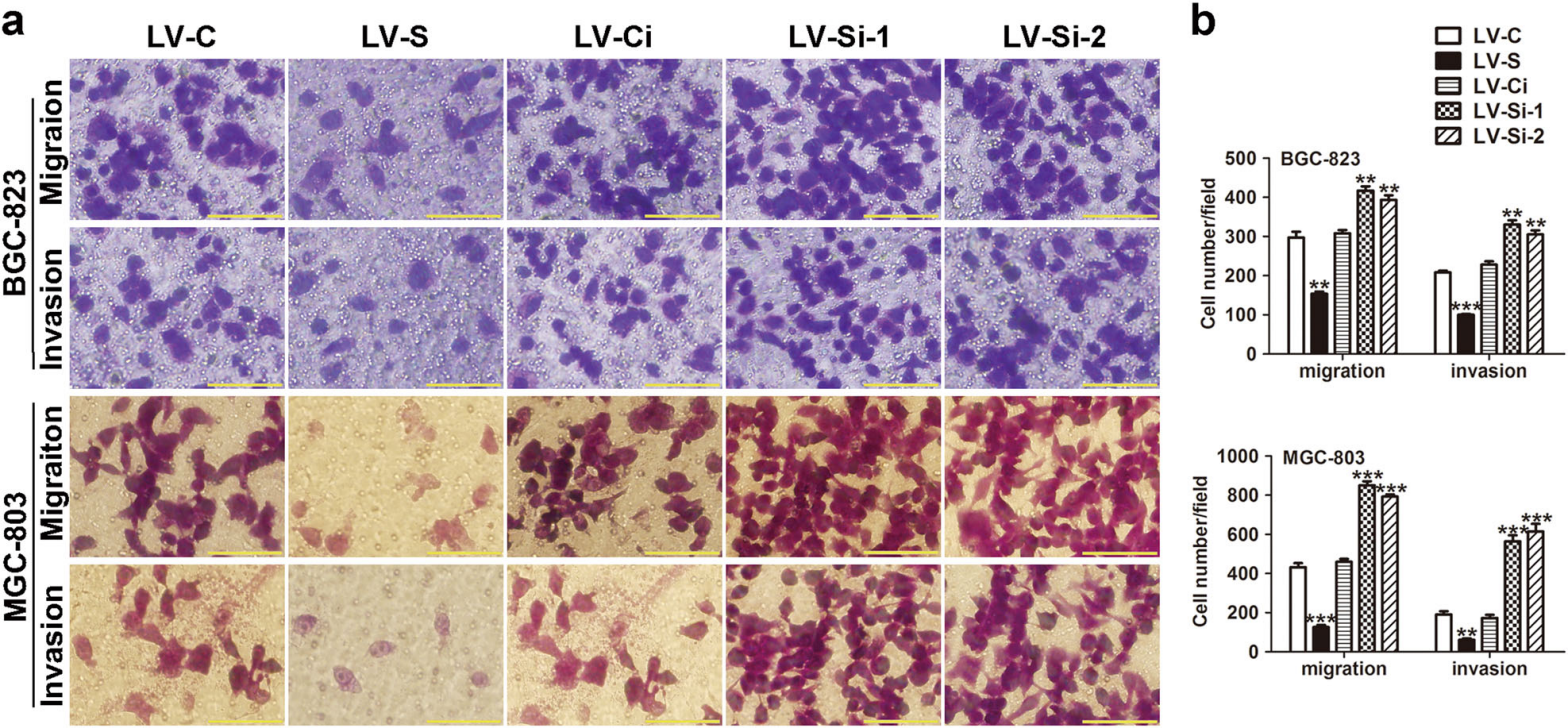
SIRT1 inhibits GC cell migration and invasion in vitro. **a** Cell migration and invasion were confirmed by Transwell assay without or with Matrigel in stably lentivirus-infected BGC-823 and MGC-803 cells. Scale bars, 100 µm. **b** Statistical analyses of migrating or invasive cells per visual field. Data indicate the mean ± SD of three independent experiments. **p < 0.01, ***p < 0.001

To evaluate the effect of SIRT1 on GC cell metastasis, stably lentivirus-infected GC cell lines AGS, BGC-823, MGC-803, HGC-27, and SGC-7901 were studied. Cells stably transfected with the SIRT1-lentiviral expression vector or its control were regarded as LV-S and LV-C cells, respectively. Cells stably transfected with the SIRT1-lentiviral shRNA or its control were regarded as LV-Si and LV-Ci cells, respectively. The wound healing assay was carried out to detect the migration of GC cells. The results showed that forced expression of SIRT1 suppressed whereas knockdown of SIRT1 promoted migration of GC cells at both time points (Supplementary Fig. 1). The suppressive effect of SIRT1 on GC cell migration was confirmed by a Transwell assay (without Matrigel; Fig. 1 and Supplementary Fig. 2). Furthermore, we assessed the invasion process using Transwells precoated with Matrigel. We found that overexpression of SIRT1 inhibited the invasive abilities of GC cells, and on the contrary, silencing of SIRT1 facilitated the invasion process (Fig. 1). Taken together, our results indicated an inhibitory effect of SIRT1 on migration and invasion of GC cells in vitro.

### SIRT1 inhibits metastasis of GC in vivo

**Fig. 2:**
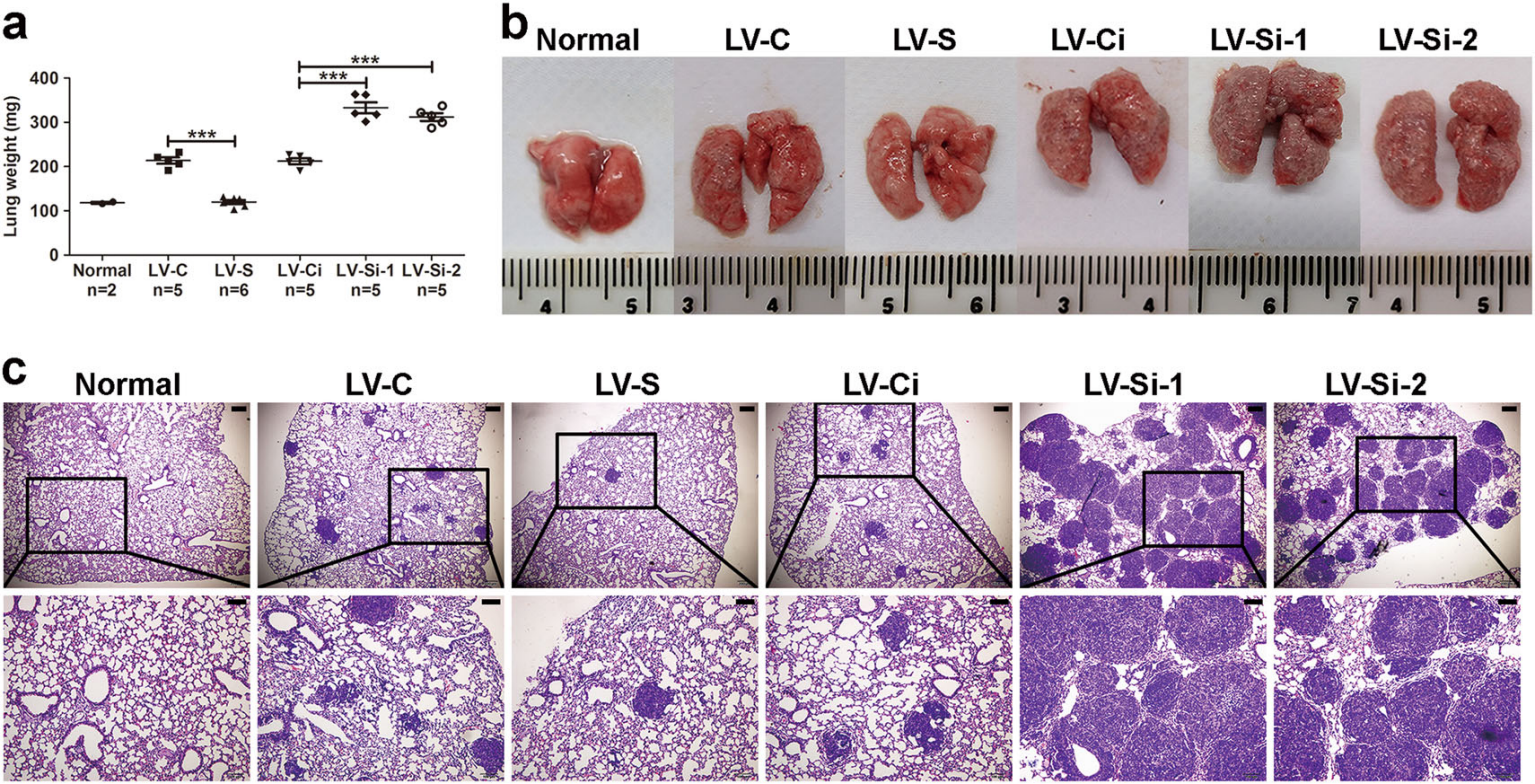
SIRT1 inhibits GC metastasis in vivo. **a** The weight of lungs was evaluated. Data indicate the mean ± SD. ***p < 0.001. **b** Representative pictures of lungs from each group. **c** HE staining of lung tissues with metastatic nodules. Scale bars, 200 µm in the upper panel and 100 µm in the lower panel

Next, we wondered whether SIRT1 inhibits tumor metastasis in vivo. Stably lentivirus-infected BGC cells of each group (5 × 10^5^ cells/mouse) were injected into immunodeficient nude mice via tail vein. Four weeks later, the mice were euthanized, and the lungs were excised for metastasis examination. The lungs from mice injected with SIRT1-forced-expression BGC-823 cells were much smaller and weighed less than their controls (Fig. 2a, b). The metastatic tumors in lungs were confirmed by HE staining of tissue slices. Fewer metastatic nodes were found in the lungs from mice with ectopic expression of SIRT1 in BGC-823 cells (Fig. 2c). On the contrary, the lungs from mice injected with SIRT1-knockdown BGC-823 cells were obviously larger and heavier than their controls (Fig. 2a, b). Moreover, BGC-823 cells with stable depletion of SIRT1 produced more metastases in the lungs of nude mice than their control cells did (Fig. 2c). Therefore, the above data suggested that SIRT1 can inhibit metastasis of GC in vivo.

### Identification of targets regulated by SIRT1 in GC cells by mRNA microarray profiling

To screen the genes that are regulated by SIRT1 in GC cells, we performed mRNA microarray profiling of the four types of stably lentivirus-infected BGC-823 cells (LV-C, LV-S, LV-Ci, and LV-Si). The microarray data were submitted to Gene Expression Omnibus (GEO, GSE103519). We obtained 46 SIRT1-positively-regulated genes and 26 SIRT1-negatively-regulated genes with no less than two-fold changes. Among these genes, those that perform some functions in tumor-related pathways were selected to confirm the expression changes. The mRNA levels were first detected in stably lentivirus-infected BGC cells, and seven of the selected genes showed significant changes in both LV-S and LV-Si groups when compared with their corresponding controls (Supplementary Fig. 3a, b). After that, we performed qPCR assays on stably lentivirus-infected AGS and MGC-803 cells, and this time, only the changes of *ARHGAP5* matched the results of the microarray analysis (Supplementary Fig. 3a, b). Furthermore, the protein levels of ARHGAP5 were consistent with mRNA expression, which was repressed by SIRT1 (Supplementary Fig. 3c). Therefore, *ARHGAP5* was chosen as the target of SIRT1 for further research.

### SIRT1 inhibits expression of ARHGAP5 by physically interacting with and deacetylating c-JUN

**Fig. 3:**
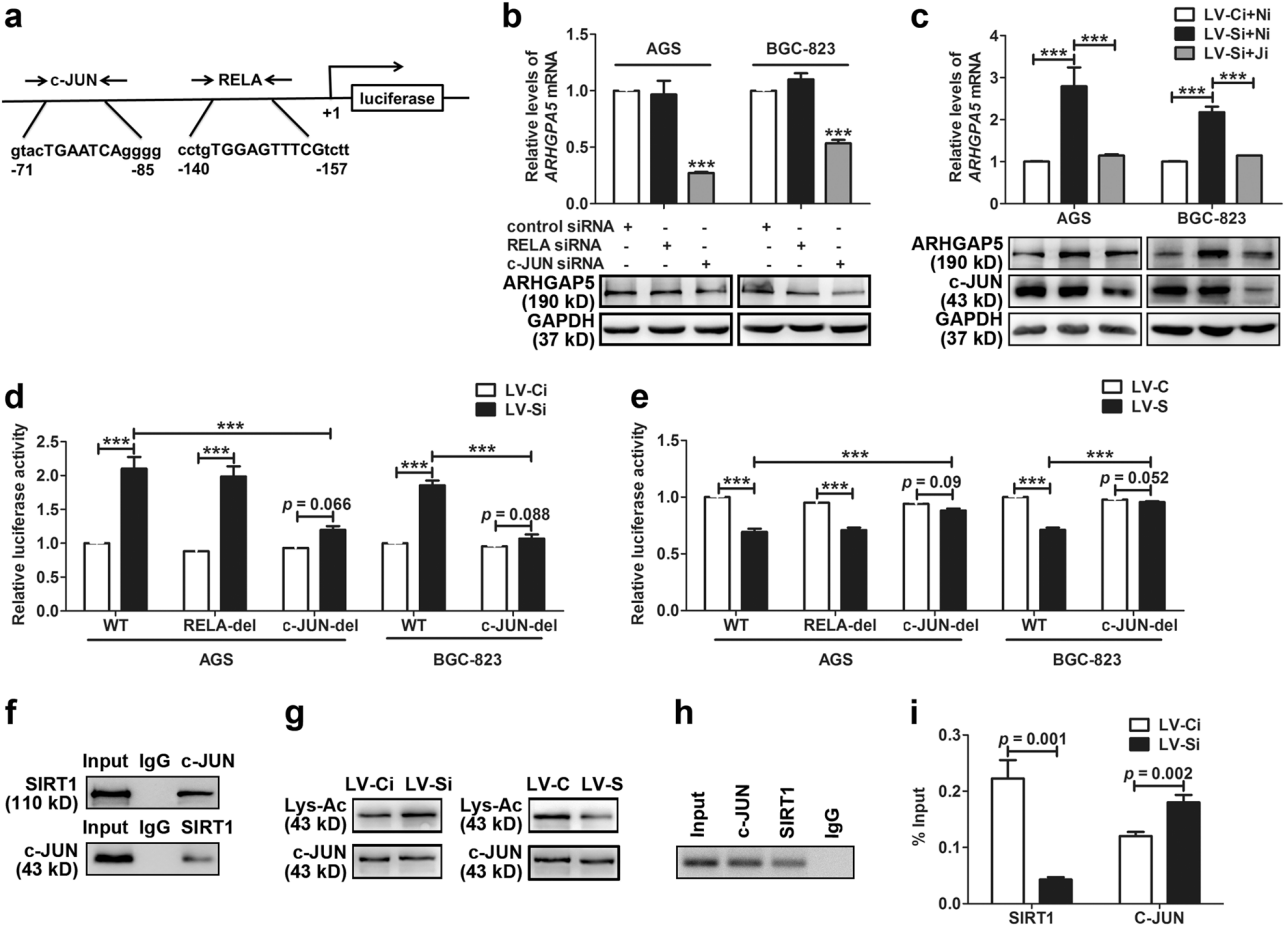
SIRT1 inhibits expression of ARHGAP5 by physically interacting with and deacetylating c-JUN. **a** The scheme of the putative c-JUN and RELA-binding sites in the ARHGAP5 promoter region. **b** Levels of ARHGAP5 in AGS and BGC-823 cells transfected with the indicated siRNAs. **c** Levels of ARHGAP5 in stably lentivirus-infected AGS and BGC-823 cells transfected with the indicated siRNAs. Ni for control siRNA and Ji for siRNA targeting c-JUN. **d,** **e** Dual luciferase activities of different ARHGAP5 promoter constructs in stably lentivirus-infected AGS and BGC-823 cells with SIRT1 knockdown (**d**) or ectopic SIRT1 expression (**e**), respectively. **f** Physical interaction between SIRT1 and c-JUN. Upper panel: AGS cell lysates were immunoprecipitated with an anti-c-JUN antibody and detected using an anti-SIRT1 antibody. Bottom panel: AGS cell lysates were immunoprecipitated with an anti-SIRT1 antibody and detected using an anti-c-JUN antibody. **g** SIRT1 deacetylates c-JUN. Lysates from stably lentivirus-infected AGS cells with the SIRT1 knockdown (left lanes) or ectopic SIRT1 expression (right lanes) were immunoprecipitated with an anti-c-JUN antibody and detected using an anti-lysine-acetylation antibody. c-JUN served as the control. **h** AGS cells were subjected to ChIP assay to analyze the occupancy of the ARHGAP5 promoter by SIRT1 at the c-JUN binding site. **i** ChIP-qPCR was performed in stably lentivirus-infected AGS cells with SIRT1 knockdown. All data indicate the mean ± SD of three independent experiments. ***p < 0.001

Then, we examined the molecular mechanism by which SIRT1 suppressed *ARHGAP5* expression. Some studies revealed that SIRT1 can deacetylate and regulate transcriptional activities of transcription factors and thus influence the expression of their targets^8,9,12,20^. Therefore, we analyzed the promoter of *ARHGAP5* in the JASPAR database and found putative binding sites of c-JUN and RELA (Fig. 3a). The siRNAs targeting the two above-mentioned transcription factors were transfected into GC cells to determine which transcription factor is involved in SIRT1’s regulation of *ARHGAP5*. After *c-JUN* siRNA transfection, knockdown of *c-JUN* and its target genes was verified by qPCR (Supplementary Fig. 4a). The efficient silencing of *c-JUN* at the protein level was confirmed by western blot (Supplementary Fig. 4b). For *RELA*, we used the validated siRNA described before^15^. Knockdown of *c-JUN* but not *RELA* downregulated *ARHGAP5* in AGS and BGC-823 cells indicating that c-JUN participates in the regulation of *ARHGAP5* expression in GC cells (Fig. 3b). Furthermore, in stably lentivirus-infected GC cells, upregulation of *ARHGAP5* induced by SIRT1 depletion was reversed by knockdown of *c-JUN* (Fig. 3c). These data suggested that c-JUN participated in the regulation of *ARHGAP5* expression by SIRT1.

To further clarify whether c-JUN is involved in SIRT1-regulated transcription of *ARHGAP5*, we performed luciferase assays. First, we transfected stably lentivirus-infected GC cells with a luciferase reporter vector containing the primary *ARHGAP5* promoter (from position −1232 to +193; regarded as WT). The results revealed that SIRT1 inhibited the luciferase activity driven by the *ARHGAP5* promoter (Fig. 3d, e). This result confirmed that SIRT1 suppressed *ARHGAP5* expression at the transcriptional level. Next, we assessed the luciferase activity driven by a mutant luciferase reporter vector in which the putative binding sites of RELA (designated as RELA-del) or c-JUN (designated as c-JUN-del) were inactivated by deletion. In stably lentivirus-infected AGS cells with SIRT1 silencing, the increase in luciferase activity diminished when they are transfected with the c-JUN-del reporter, not the RELA-del reporter (Fig. 3d). On the contrary, the decrease in luciferase activity was reversed by the c-JUN-del reporter, not the RELA-del reporter, in AGS cells with ectopic SIRT1 expression (Fig. 3e). Moreover, a failure of SIRT1’s regulation of the activity of the *ARHGAP5* promoter with a putative c-JUN binding-site deletion was also detected in stably lentivirus-infected BGC-823 cells (Fig. 3d, e). These findings indicated that c-JUN is involved in SIRT1-driven repression of ARHGAP5.

It has been shown that SIRT1 can interact with and deacetylate c-JUN, thus yielding an inactive transcription factor^20,21^. To determine whether SIRT1 interacts with c-JUN in GC cells, we conducted co-IP experiments. As shown in Fig. 3f, in AGS cells, the SIRT1 signal was detected in the protein complex immunoprecipitated with c-JUN, and a signal of c-JUN was detectable in the protein complex immunoprecipitated with SIRT1. This result indicated a physical interaction between SIRT1 and c-JUN. Furthermore, acetylation levels of c-JUN increased with SIRT1 silencing, suggesting that SIRT1 not only physically interacts with but also deacetylates c-JUN (Fig. 3g). Next, we tested whether SIRT1 can be recruited to the *ARHGAP5* promoter at the c-JUN-binding site. ChIP assays were performed, and the results showed that SIRT1 occupied the *ARHGAP5* promoter at the same site as c-JUN did (Fig. 3h). As predicted, sharp reduction of SIRT1 and significant accumulation of c-JUN at the *ARHGAP5* promoter were observed in stable SIRT1-knockdown AGS cells (Fig. 3i). Taken together, these data suggested that SIRT1 physically interacted with c-JUN, was recruited to the *ARHGAP5* promoter, deacetylated c-JUN, and thus suppressed transcriptional activity of c-JUN and expression of ARHGAP5.

### Enhanced ARHGAP5 expression correlates with GC progression

**Fig. 4:**
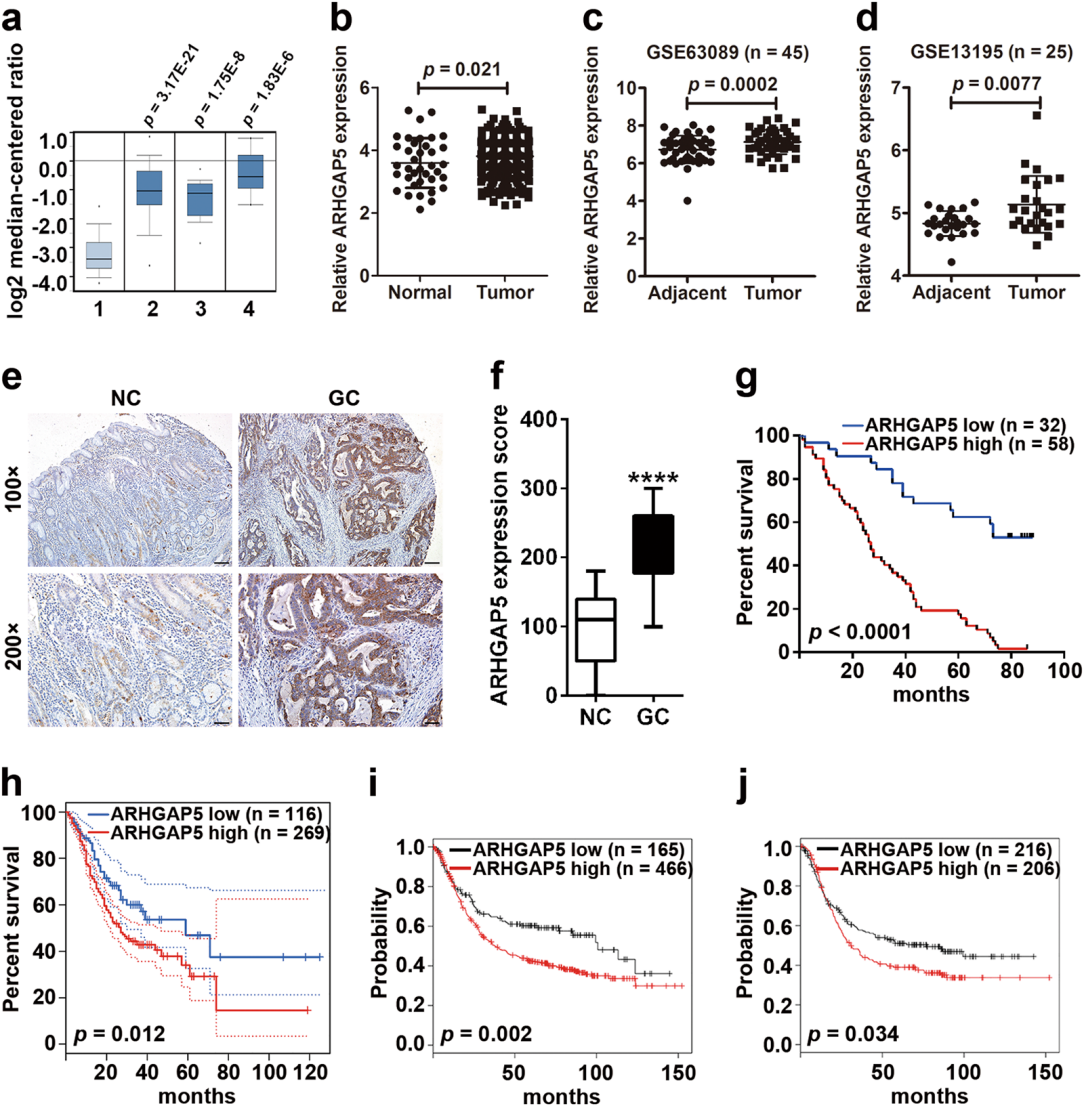
Enhanced ARHGAP5 expression correlates with GC progression. **a–f** Detection of ARHGAP5 levels in GC and normal tissues using Oncomine database (**a**), TCGA data set (**b**), NCBI GEO repository (**c, d**), and tissue array (**e, f**). In the results from the Oncomine database, 1 represents Normal Gastric Mucosa (*n* = 29), 2 denotes Gastric Intestinal Type Adenocarcinoma (*n* = 65), 3 represents Diffuse Gastric Adenocarcinoma (*n* = 13), and 4 stands for Gastric Mixed Adenocarcinoma (*n* = 8). The *p*-values were obtained from the Oncomine database. **g–j** Analysis of ARHGAP5 levels in relation to the overall survival of patients with GC. **g** Tissue array, scale bar: 100 µm for the upper panel, 50 µm for the bottom panel, **h** TCGA data set, and **i, j** Kaplan–Meier Plotter database. In the results from Kaplan–Meier Plotter database, overall survival analysis of GC patients with lymph node metastasis was shown in **j**. The data and *p*-values were obtained from the Kaplan–Meier Plotter database (235635_at). All data indicate the mean ± SD. ****p < 0.0001

Recently, ARHGAP5 was identified as an oncogene that promotes tumor metastasis^22–24^. To explore the possible participation of ARHGAP5 in the progression of GC, we analyzed the Oncomine database and found that *ARHGAP5* levels are higher in different types of GC (Fig. 4a). Search results from the TCGA data set also showed that *ARHGAP5* was significantly upregulated in GC (Fig. 4b). Unfortunately, when we compared the 34 pairs of samples of tumors and their corresponding adjacent normal tissues, we observed no obvious difference in the expression of *ARHGAP5* (*p* = 0.4107, data not shown). By contrast, data from the NCBI GEO database (GSE63089 and GSE13195) indicated higher *ARHGAP5* levels in GC tissues compared to their corresponding adjacent normal mucosa (Fig. 4c, d). To determine the protein levels of ARHGAP5, we carried out IHC staining using the tissue array including 90 pairs of paraffin-embedded GC and matched normal tissues. Staining results showed that ARHGAP5 expression was low in non-cancerous gastric mucosa with four of which no staining at all (Fig. 4e, f). But in GC tissues, levels of ARHGAP5 were significantly enhanced (Fig. 4e, f). The above data prove that ARHGAP5 is markedly upregulated in GC.

Next, we analyzed whether the expression of ARHGAP5 was correlated with the clinicopathological characters and patients’ survival. The data showed that ARHGAP5 expression was positively correlated with tumor size (*p* = 0.003), depth of tumor infiltration (T stage, *p* < 0.001), local lymph node metastasis (N stage, *p* = 0.003), and clinical stage (TNM stage, *p* < 0.001) (Supplementary Table 2). Using univariate Cox regression analyses, we found that tumor size (*p* = 0.003), depth of tumor infiltration (T stage, *p* = 0.004), local lymph node metastasis (N stage, *p* = 0.002), clinical stage (TNM stage, *p* = 0.002), and ARHGAP5 levels (*p* < 0.001) were significantly associated with patients’ survival. Furthermore, multivariate Cox regression analysis further confirmed the depth of tumor infiltration (T stage, *p* = 0.013), local lymph node metastasis (N stage, *p* = 0.008), and ARHGAP5 levels (*p* = 0.001) as independent predictors of the overall survival of GC patients (Supplementary Table 3).

Then, survival curves were plotted to compare patients’ outcomes according to the expression levels of ARHGAP5. The overall survival of GC patients with high ARHGAP5 levels was markedly worse than that of GC patients with low ARHGAP5 expression (Fig. 4g). Analysis of GC samples in TCGA database showed that overall survival periods are shorter among patients with higher ARHGAP5 levels in the tumor (Fig. 4h). Data from Kaplan–Meier plotter also indicated that lower expression of ARHGAP5 in the tumor results in a longer survival period (Fig. 4i). Furthermore, when we included lymph node metastasis in overall survival analysis, the influence of ARHGAP5 remained significant (Fig. 4j). Taken together, above results uncovered a potential link between increased ARHGAP5 levels and GC progression.

### ARHGAP5 is involved in SIRT1-induced suppression of GC cell migration and invasion in vitro and metastasis in vivo

**Fig. 5:**
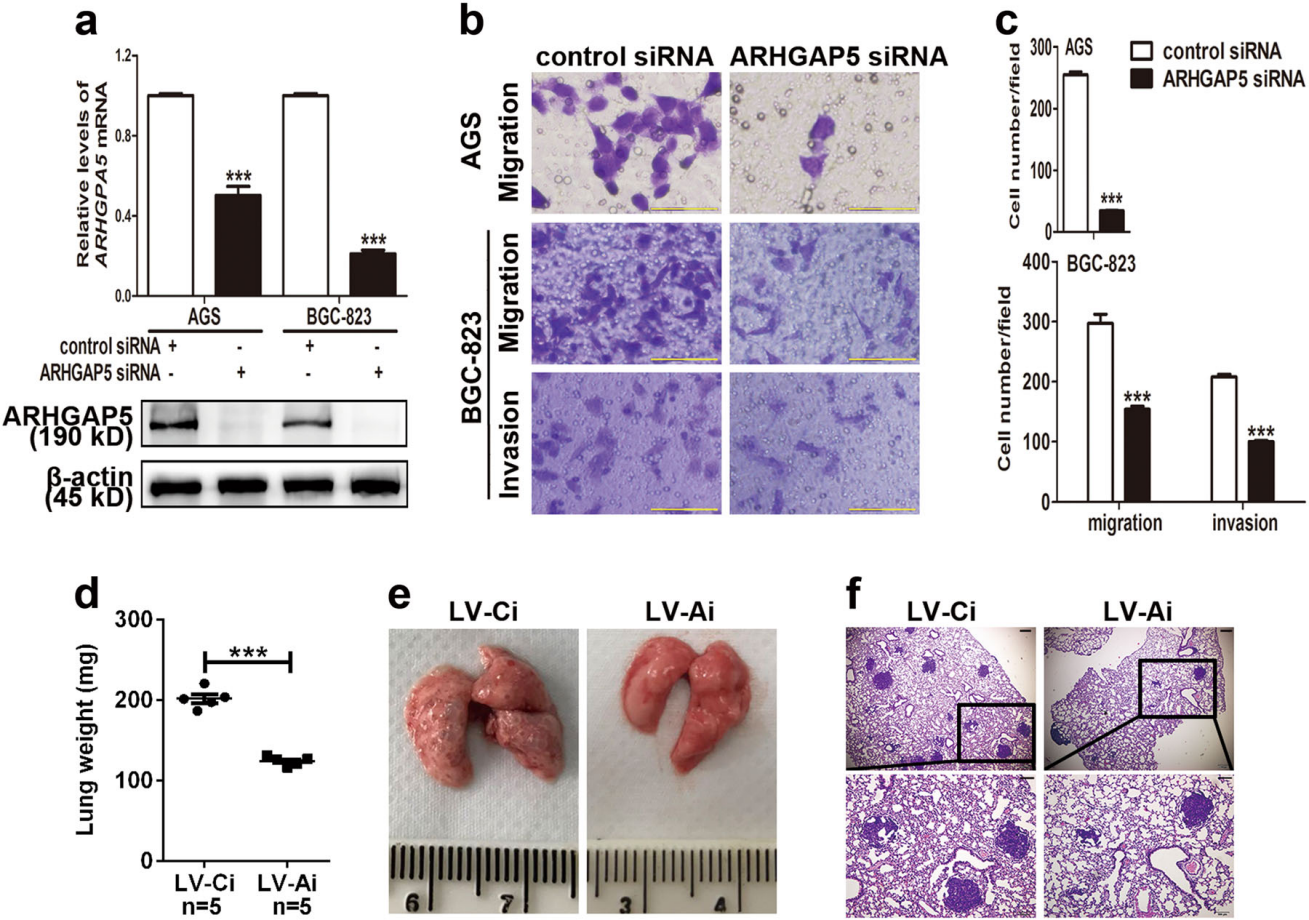
ARHGAP5 downregulation suppresses migration, invasion, and metastasis of GC in vitro and in vivo. **a** Effective knockdown of ARHGAP5 by siRNA was examined by qPCR and western blot. **b** Cell migration and invasion were evaluated by Transwell assays without or with Matrigel in AGS and BGC-823 cells transfected with the indicated siRNAs. Scale bars, 100 µm. **c** Statistical analyses of migrating or invasive cells per visual field. **d** The weight of lungs was evaluated. **e** Representative pictures of lungs from each group. **f** HE staining of lung tissues with metastatic nodules. Scale bars, 200 µm in the upper panel and 100 µm in the lower panel. All data indicate the mean ± SD. ***p < 0.001

Next, we determined whether ARHGAP5 plays a role in the migration and invasion of GC cells. Specific siRNAs targeting *ARHGAP5* were transfected into GC cells and dramatically reduced expression levels of ARHGAP5 at both mRNA and protein levels (Fig. 5a). Transwell assays without or with Matrigel indicated that cell migration and invasion were significantly suppressed via downregulation of ARHGAP5 (Fig. 5b, c). To evaluate the effect of ARHGAP5 on GC cell metastasis in vivo, BGC-823 cells were stably infected with ARHGAP5-lentiviral shRNA. Stably knockdown of ARHGAP5 in BGC-823 cells was validated at both mRNA and protein levels (Supplementary Fig. 5a and c). Then nude mice were used to determine the lung metastasis of tail vein-injected GC cells. At the end of in vivo metastasis experiment, the lungs from mice injected with ARHGAP5-knockdown BGC-823 cells showed less weight, smaller size, and fewer metastatic nodes than their controls (Fig. 5d–f). Therefore, ARHGAP5 acts as an oncogene promoting GC cell migration and invasion in vitro and metastasis in vivo.

**Fig. 6:**
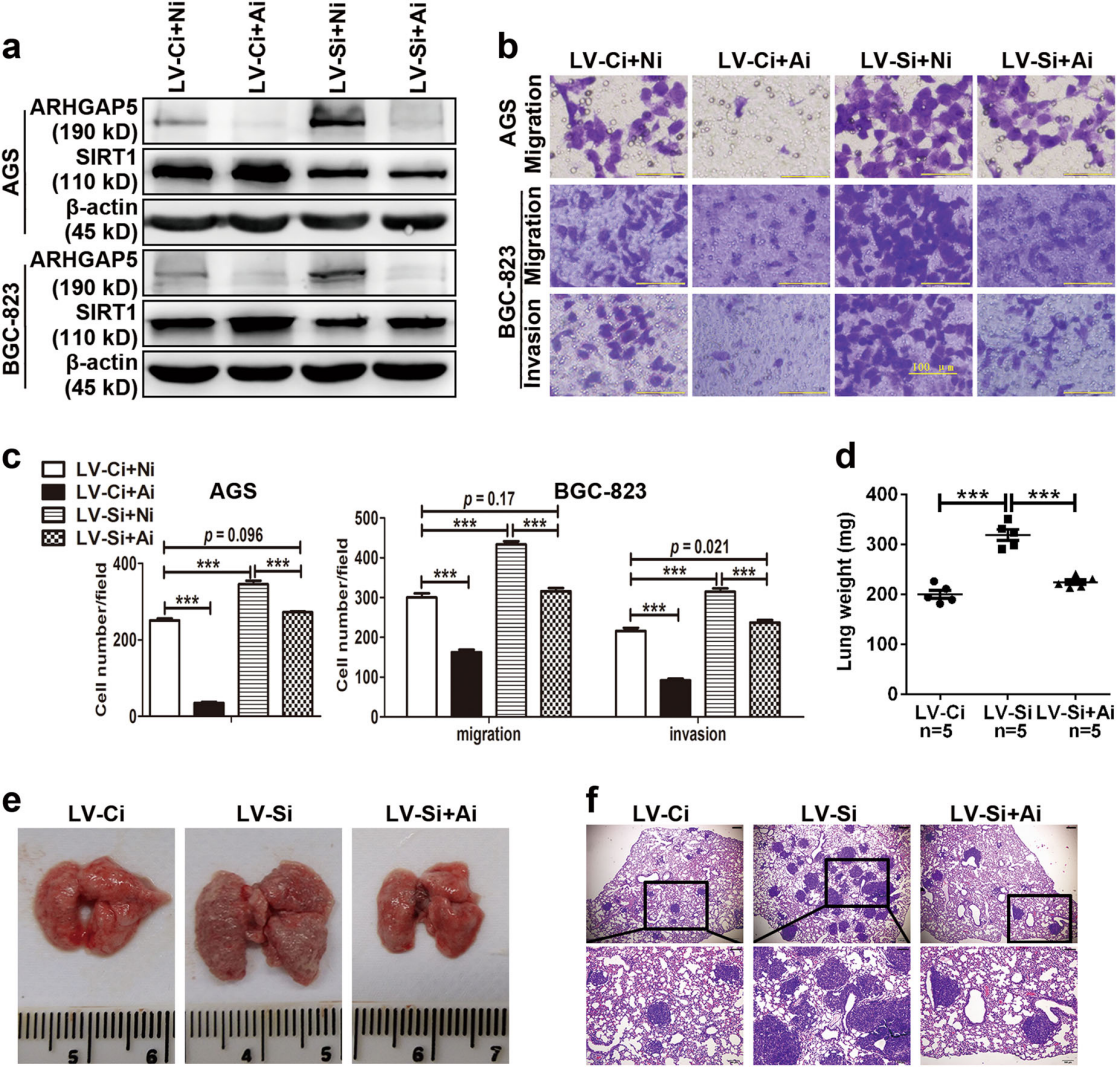
ARHGAP5 is involved in SIRT1-induced suppression of GC cell migration and invasion. **a** Effective knockdown of ARHGAP5 in stably lentivirus-infected AGS and BGC-823 cells with SIRT1 depletion and their controls. Ni for control siRNA and Ai for siRNA targeting ARHGAP5. **b** Cell migration and invasion were evaluated by Transwell assays without or with Matrigel in stably lentivirus-infected AGS and BGC-823 cells (LV-Ci and LV-Si) transfected with the indicated siRNAs. Scale bars, 100 µm. The statistical analyses of migrating or invasive cells per visual field were presented as histogram in **c**. **d** The weight of lungs was evaluated. **e** Representative pictures of lungs from each group. **f** HE staining of lung tissues with metastatic nodules. Scale bars, 200 µm in the upper panel and 100 µm in the lower panel. All data indicate the mean ± SD. ***p < 0.001

In addition, to determine whether dysregulation of ARHGAP5 is involved in the suppression of cell migration and invasion by SIRT1, we transfected siRNAs against *ARHGAP5* into stably SIRT1-silenced GC cells. The effective knockdown of ARHGAP5 was validated (Fig. 6a). Of note, we found that downregulation of ARHGAP5 significantly suppressed cell migration and invasion of GC cells and clearly reversed the promotion of migration and invasion induced by SIRT1 depletion (Fig. 6b, c). Furthermore, the in vivo lung metastasis model was set up using nude mice. Besides stably SIRT1-silencing BGC-823 cells and the control, stably SIRT1-silencing BGC-823 cells with ARHGAP5 knockdown by lentivirus were also injected. As expected, levels of ARHGAP5 were effectively restored in stably SIRT1-silencing BGC-823 cells with ARHGAP5 knockdown (Supplementary Fig. 5b, c). The enhanced cell metastasis to the lung induced by SIRT1 silencing was also reversed by knockdown of ARHGAP5 (Fig. 6d–f). Taken together, both the in vitro and in vivo results provided evidence that ARHGAP5 is a functional target of SIRT1 in GC metastasis.

## Discussion

Dysregulated expression of SIRT1 is a frequent molecular event in human cancers^15,25–27^. Our previous study has shown that SIRT1 is downregulated in GC and inhibits GC cell proliferation and xenografted tumor growth. Nevertheless, the role of SIRT1 in progression and metastasis of GC is still largely unknown. Here, we provide evidence that SIRT1 acts as a critical negative regulator of metastasis of GC. Through in vitro and in vivo SIRT1 gain or loss-of-function experiments, we found that SIRT1 suppressed migration and invasion of GC cells and diminished lung metastasis of GC. Recent analyses involving gene expression data of GC patients derived from the NCBI GEO repository indicate that SIRT1 serves as a powerful prognostic marker of GC, and higher expression of SIRT1 correlates with longer overall survival and first progression periods^28,29^. Besides, data from the Kaplan–Meier Plotter database (218878_s_at) show that increased SIRT1 levels result in better overall survival. The median survival period for patients in the high-SIRT1-expression cohort is 36.4 months but 22.3 months for patients with low SIRT1 levels (data not shown). These results are suggestive of a tumor suppressive action of SIRT1 in GC progression, in agreement with our data. Nonetheless, the molecular mechanisms of SIRT1’s inhibition of progression and metastasis of GC have yet to be elucidated.

In this report, we employed mRNA microarray profiling to screen the target genes that are regulated by SIRT1. Considering that the difference should be significant among different groups (SIRT1 overexpression or knockdown) and universal in different types of GC cells, we found that *ARHGAP5* is downregulated by SIRT1. Because there are few studies on the transcriptional regulation of *ARHGAP5*, we analyzed the promoter region of *ARHGAP5* by searching for the transcription factors with which SIRT1 usually cooperates. One putative binding site was found for both RELA and c-JUN. Silencing of *c-JUN*, but not *RELA*, decreased the expression of ARHGAP5 in GC cells. The luciferase activity assay confirmed that it is c-JUN, not RELA, which is involved in SIRT1-induced transcriptional repression of *ARHGAP5*. It has been reported that SIRT1 can directly inhibit the transcriptional activity of c-JUN and expression of its targets and thereby exerts a suppressive effect on cell proliferation and migration^21,30^. Our co-IP assays indicate that SIRT1 physically interacts with and deacetylates c-JUN in GC cells. Furthermore, results from ChIP experiments confirmed SIRT1’s association with c-JUN on the *ARHGAP5* promoter and the inhibitory action of SIRT1 on c-JUN’s binding to this promoter.

ARHGAP5, a prototypical member of the family of Rho GTPase-activating proteins (GAPs), is a potent Rho regulator^31^. Therefore, it is not surprising that ARHGAP5 is involved in the process of cellular motility. Nakahara et al.^32^ have reported that ARHGAP5 is associated with F-actin in invadopodia and promotes membrane-protrusive and degradative activities, which are necessary for cell invasion. In human umbilical vein endothelial cells, absence of ARHGAP5 results in a significant reduction in the levels of matrix metalloproteinases and matrix degradation^33^. Moreover, emerging evidence shows that ARHGAP5 is upregulated in cancers and contributes to invasive and metastatic behavior. By enhancing the migration, invasion, and metastasis, ARHGAP5 reveals its protumorigenic functions in a variety of cancers, including breast cancer^22^, non-small cell lung cancer^23^, nasopharyngeal carcinoma^24^, and hepatocellular carcinoma^34^. In line with the previous findings, experiments and analyses presented here reveal an increased level of ARHGAP5 in GC and that high expression of ARHGAP5 in GC leads to poor prognosis. Silencing of ARHGAP5 significantly restrained GC cell migration and invasion in vitro and metastasis in vivo. This finding points to the oncogenic role of ARHGAP5 in GC. Furthermore, our functional analysis showed that knockdown of ARHGAP5 in GC almost completely reversed the enhancement of migration, invasion, and metastasis induced by SIRT1 depletion. These observations confirm definitive involvement of ARHGAP5 in SIRT1-induced suppression of GC migration and invasion.

**Fig. 7:**
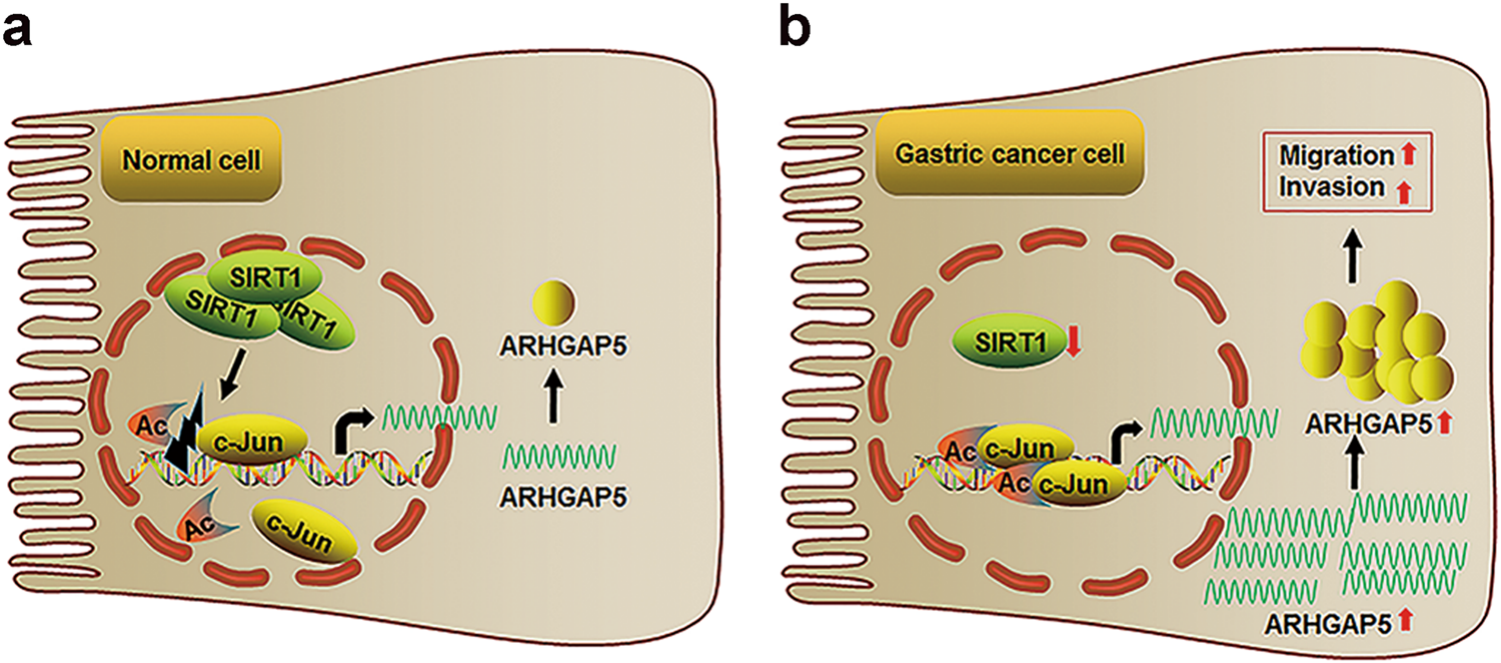
Working model for SIRT1–c-JUN–ARHGAP5 axis-mediated cell migration and invasion in gastric cancer. **a** Normal gastric epithelial cells and **b** gastric cancer cells

In summary, we have demonstrated that SIRT1 acts as a tumor suppressor in terms of invasion and metastasis of GC. SIRT1 exerts these inhibitory effects by targeting ARHGAP5 via an interaction with deacetylation and inhibition of c-JUN (Fig. 7). Therefore, the SIRT1–c-JUN–ARHGAP5 axis may represent a novel mechanism underlying the progression and metastasis of GC.

## Electronic supplementary material

Supplementary Figure Legends

Supplementary Figure 1

Supplementary Figure 2

Supplementary Figure 3

Supplementary Figure 4

Supplementary Figure 5

Supplemetary Table 1

Supplementary Table 2

Supplementary Table 3
