# Supplementary material for: SIRT1 suppresses the migration and invasion of gastric cancer by regulating ARHGAP5 expression
Source: Cell Death Dis. 2018 Sep 24;9(10):977. doi: 10.1038/s41419-018-1033-8 (PMC6155157; doi:10.1038/s41419-018-1033-8)
Supplement: Supplementary file 1 — Supplementary Figure Legends [file 41419_2018_1033_MOESM1_ESM.docx]

**Supplementary Fig. 1. SIRT1 inhibits GC cell migration (wound healing assay).** **a-b** Wound healing assay was performed to evaluate migration of stably lentivirus-infected GC cells. Scale bars, 200 µm. **c-e** Statistical analyses of migration ratios. Data indicate the mean ± SD of three independent experiments. ** *p* < 0.01, *** *p* < 0.001.

**Supplementary Fig. 2. SIRT1 inhibits GC cell migration (Transwell assay).** **a** Cell migration was confirmed by Transwell assays without Matrigel in stably lentivirus-infected AGS, HGC-27, and SGC-7901 cells. Scale bars, 100 µm. **b** Statistical analyses of migrating cells per visual field. Data indicate the mean ± SD of three independent experiments. ** *p* < 0.01, *** *p* < 0.001.

**Supplementary Fig. 3. Detection of the levels of differentially expressed genes identified by microarray.** qPCR was conducted to examine the mRNA levels of selected genes in stably lentivirus-infected GC cell lines AGS, BGC-823, and MGC-803. Cells with SIRT1 overexpression (**a**) or silencing (**b**) were studied. The genes showing changes consistent with the results of the microarray were indicated by “#”. All data indicate the mean ± SD of three independent experiments. **c**, Western blot was performed to examine the protein levels of ARHGAP5 in stably lentivirus-infected GC cell lines AGS and BGC-823.

**Supplementary Fig. 4. Effective knockdown of *c-JUN* with siRNA.** After transfection of the indicated siRNAs into GC cells, qPCR (a) and western blot (b) were carried out to examine the knockdown efficiency of c-JUN. Ni for control siRNA and Ji for siRNA targeting c-JUN. Data indicate the mean ± SD of three independent experiments. ** *p* < 0.01, *** *p* < 0.001.

**Supplementary Fig. 5. Stable knockdown of *ARHGAP5*.** In BGC-823 cells stably infected with indicated lentivirus-shRNAs, qPCR (a, b) and western blot (c) were performed to examine the silencing efficiency of ARHGAP5. Ci for control shRNA, Si for SIRT1-shRNA, and Ai for ARHGAP5-shRNA. Data indicate the mean ± SD of three independent experiments. *** *p* < 0.001.
