# Supplementary figures and images for: SIRT1 suppresses the migration and invasion of gastric cancer by regulating ARHGAP5 expression
Source: Cell Death Dis. 2018 Sep 24;9(10):977. doi: 10.1038/s41419-018-1033-8 (PMC6155157; doi:10.1038/s41419-018-1033-8)

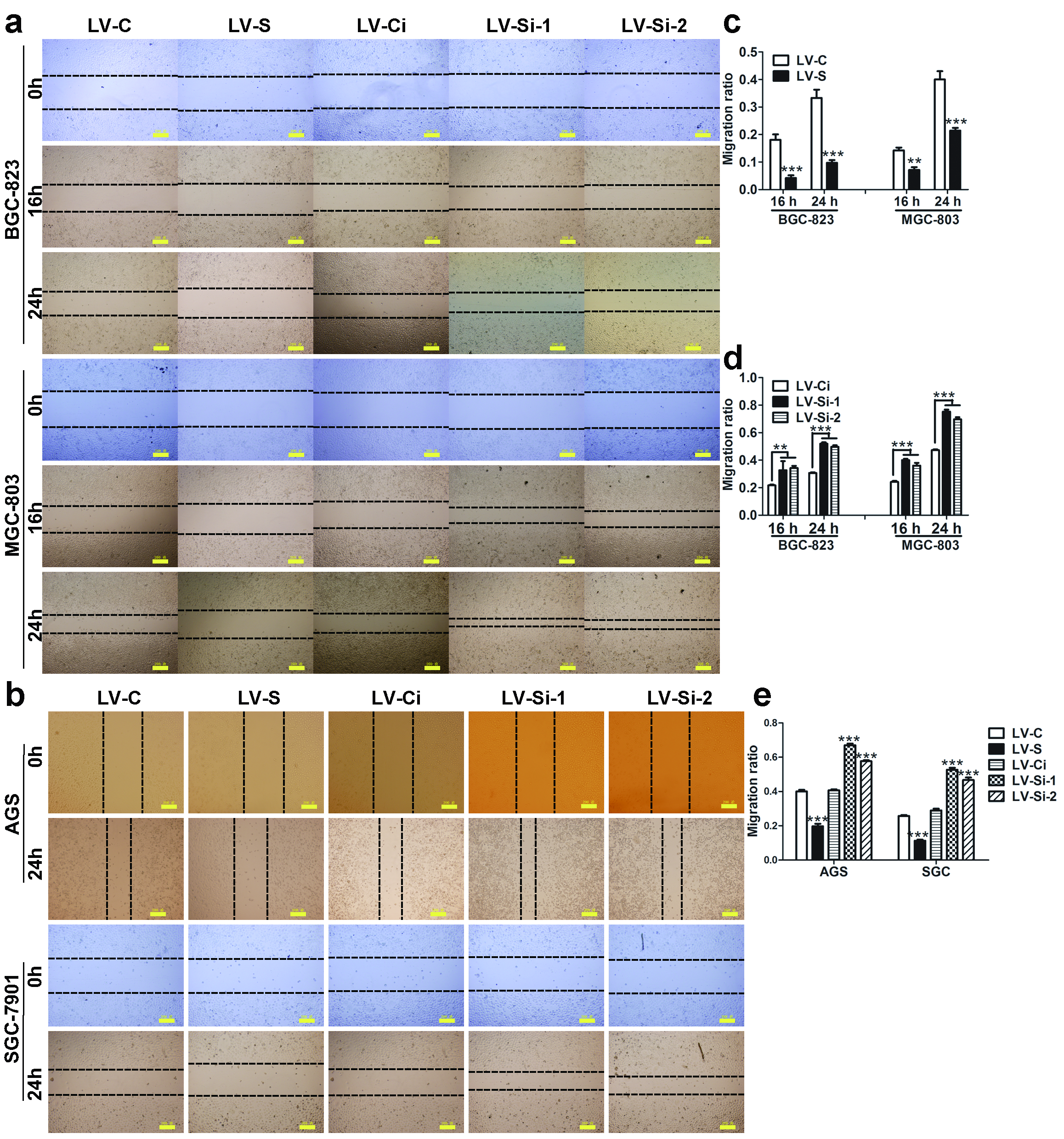

Supplement: Supplementary file 2 — Supplementary Figure 1 [file 41419_2018_1033_MOESM2_ESM.tif]

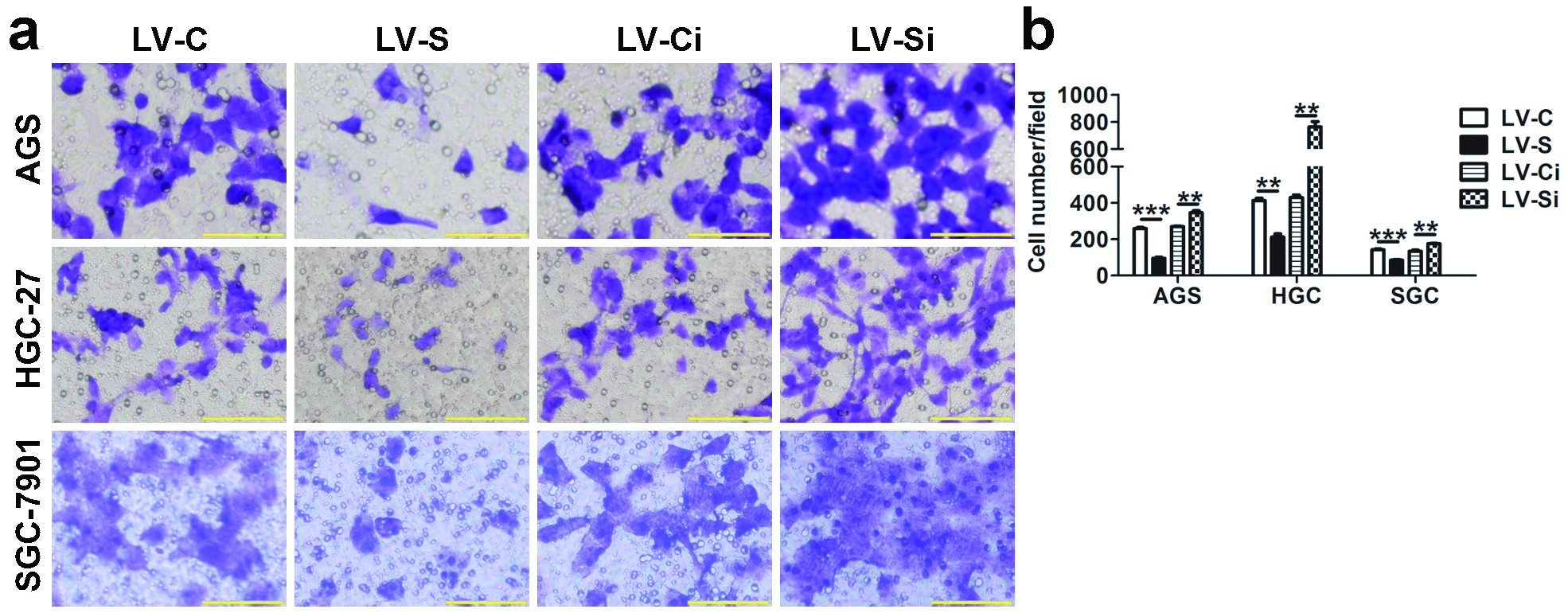

Supplement: Supplementary file 3 — Supplementary Figure 2 [file 41419_2018_1033_MOESM3_ESM.tif]

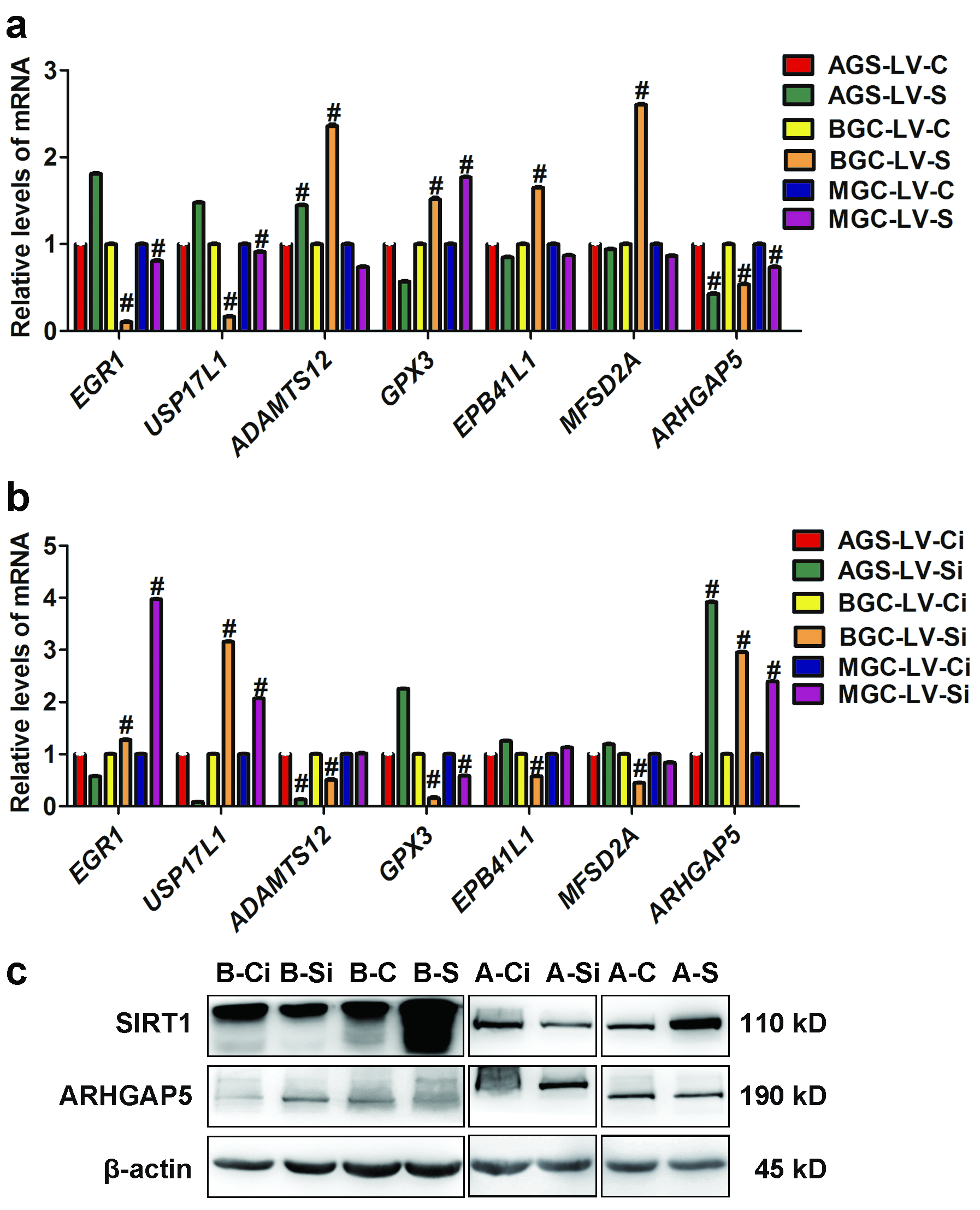

Supplement: Supplementary file 4 — Supplementary Figure 3 [file 41419_2018_1033_MOESM4_ESM.tif]

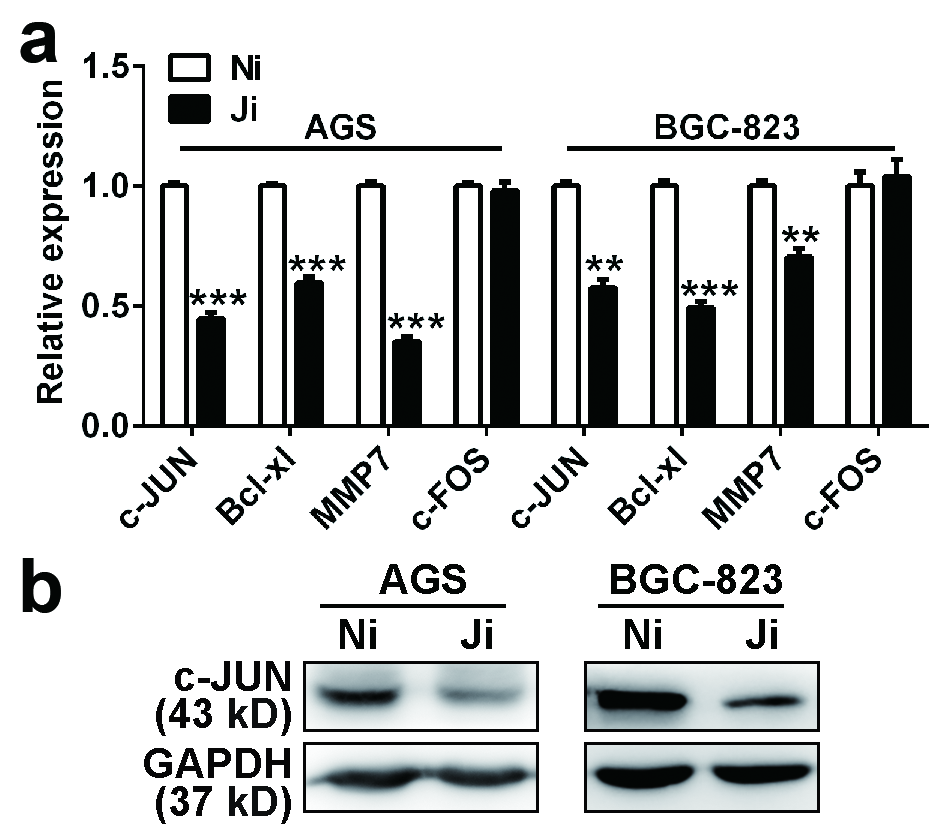

Supplement: Supplementary file 5 — Supplementary Figure 4 [file 41419_2018_1033_MOESM5_ESM.tif]

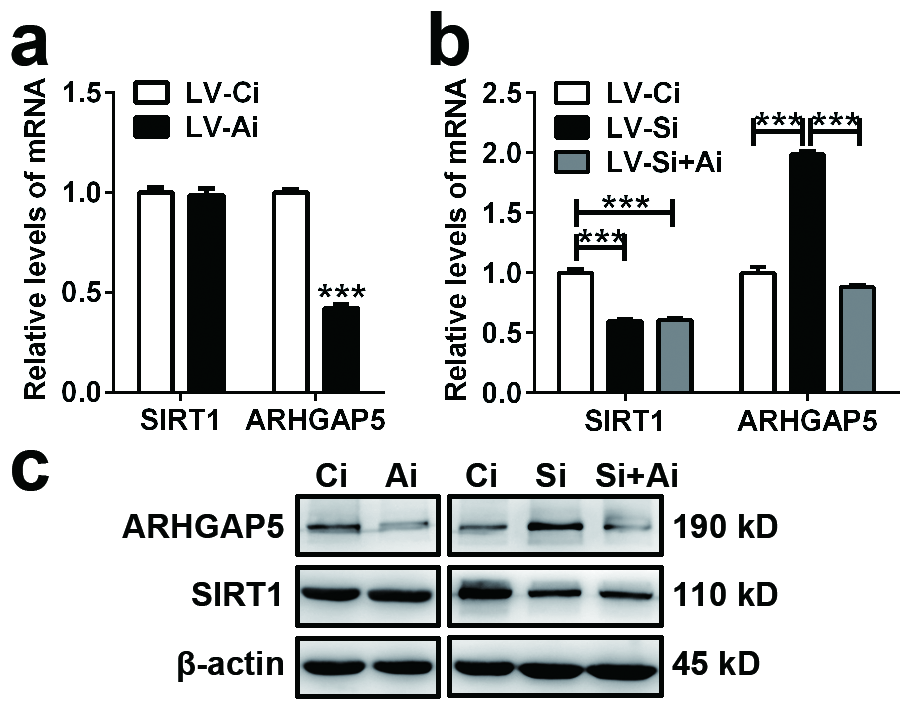

Supplement: Supplementary file 6 — Supplementary Figure 5 [file 41419_2018_1033_MOESM6_ESM.tif]
