# Supplementary material for: SIRT1 suppresses the migration and invasion of gastric cancer by regulating ARHGAP5 expression
Source: Cell Death Dis. 2018 Sep 24;9(10):977. doi: 10.1038/s41419-018-1033-8 (PMC6155157; doi:10.1038/s41419-018-1033-8)
Supplement: Supplementary file 7 — Supplemetary Table 1 [file 41419_2018_1033_MOESM7_ESM.doc]

**Supplementary Table 1. Sequences of siRNAs and primers used in this study.**

|  | **Gene** | **Sequence (5’ → 3’)** |
| --- | --- | --- |
| **siRNA** | *c-JUN* | CUGAUAAUCCAGUCCAGCATT^1,2^ |
|  | *RELA* | AGCACAGAUACCACCAAGATT |
|  | *ARHGAP5* | AGAUCAUAAUAUCAAUCUATT^3,4^ |
| **Primers** | *c-JUN* | TTGGCTTCTCGCTCCACTTT  GATCGCTCGCCAACTACAGA |
|  | *RELA* | ATGTGGAGATCATTGAGCAGC  CCTGGTCCTGTGTAGCCATT |
|  | *ARHGAP5* | AGGGAAGCTCAACGTAGATGG  ATGATCCACGCATTCATCACAT |
|  | *Bcl-xl* | ACTGTGCGTGGAAAGCGTAG  TTCCACAAAAGTATCCCAGCC |
|  | *MMP7* | GAGTGAGCTACAGTGGGAACA  CTATGACGCGGGAGTTTAACAT |
|  | *c-FOS* | CCAAGCGGAGACAGACCAAC  ATCAGGGATCTTGCAGGCAG |
|  | *β-actin* | TTGCCGACAGGATGCAGAA GCCGATCCACACGGAGTACT |
|  | *ARHGAP5* promoter  (c-JUN binding site) | TTGGCTTCTCGCTCCACTTT  GATCGCTCGCCAACTACAGA |

# ****References:****

# [**Wu**](https://www-ncbi-nlm-nih-gov.proxy.library.vanderbilt.edu/pubmed/?term=Wu H%5BAuthor%5D&cauthor=true&cauthor_uid=20847229)****, H. et al. Regulation of Nur77 expression by β-catenin and its mitogenic effect in colon cancer cells.**** [***FASEB J***](https://www-ncbi-nlm-nih-gov.proxy.library.vanderbilt.edu/pubmed/20847229)*****.*** 25**, 192-205 (2011).****

# [**Liu**](https://www-ncbi-nlm-nih-gov.proxy.library.vanderbilt.edu/pubmed/?term=Liu X%5BAuthor%5D&cauthor=true&cauthor_uid=29187213)****, X. et al. JMJD6 promotes melanoma carcinogenesis through regulation of the alternative splicing of PAK1, a key MAPK signaling component.**** [***Mol. Cancer***](https://www-ncbi-nlm-nih-gov.proxy.library.vanderbilt.edu/pubmed/?cmd=HistorySearch&querykey=1) **16**, 175 (2017).****

# [**Wang**](https://www.ncbi.nlm.nih.gov/pubmed/?term=Wang J%5BAuthor%5D&cauthor=true&cauthor_uid=23474761)****, J. et al. Downregulation of miR-486-5p contributes to tumor progression and metastasis by targeting protumorigenic ARHGAP5 in lung cancer.**** [***Oncogene***](https://www.ncbi.nlm.nih.gov/pubmed/23474761) **33**, 1181-1189 (2014).****

# [**Fang**](https://www.ncbi.nlm.nih.gov/pubmed/?term=Fang Y%5BAuthor%5D&cauthor=true&cauthor_uid=25961434)****, Y. et al. MiR-744 functions as a proto-oncogene in nasopharyngeal carcinoma progression and metastasis via transcriptional control of ARHGAP5.**** [***Oncotarget***](https://www.ncbi.nlm.nih.gov/pubmed/25961434) **6**, 13164-13175 (2015).****
