# Supplementary material for: SIRT1 suppresses the migration and invasion of gastric cancer by regulating ARHGAP5 expression
Source: Cell Death Dis. 2018 Sep 24;9(10):977. doi: 10.1038/s41419-018-1033-8 (PMC6155157; doi:10.1038/s41419-018-1033-8)
Supplement: Supplementary file 8 — Supplementary Table 2 [file 41419_2018_1033_MOESM8_ESM.docx]

**Supplementary Table 2. Correlation between ARHGAP5 expression and clinicopathological characters in gastric cancer patients.**

| **Clinicopathological characters** | **Number** | **ARHGAP5 expression** | | |
| --- | --- | --- | --- | --- |
|  |  | **Low** | **High** | ***p* value** |
| **Gender** |  |  |  | 0.556 |
| Men | 70 | 26 (37.1%) | 44 (62.9%) |  |
| Women | 20 | 6 (30.0%) | 14 (70.0%) |  |
| **Age (year)**^1^ |  |  |  | 0.66 |
| ≤ 66 | 45 | 17 (37.8%) | 28 (62.2%) |  |
| > 66 | 45 | 15 (33.3%) | 30 (66.7%) |  |
| **Tumor size (cm^3^)**^1,2^ |  |  |  | 0.003^3^ |
| ≤ 32 | 45 | 22 (48.9%) | 23 (51.1%) |  |
| > 32 | 42 | 8 (19.0%) | 34 (81.0%) |  |
| **Tumor infiltration** |  |  |  | < 0.001^3^ |
| T1/T2 | 11 | 11 (10.0%) | 0 (0.0%) |  |
| T3 | 61 | 16 (26.2%) | 45 (73.8%) |  |
| T4 | 18 | 5 (27.8%) | 13(72.2%) |  |
| **Local lymph node metastasis** |  |  |  | 0.003^3^ |
| 0 | 23 | 13 (56.5%) | 10 (43.5%) |  |
| 1 | 16 | 7 (43.8%) | 9 (56.2%) |  |
| 2 | 25 | 10 (40.0%) | 15 (60.0%) |  |
| 3 | 26 | 2 (7.7%) | 24 (92.3%) |  |
| **Distant metastasis** |  |  |  | 0.129 |
| 0 | 86 | 32 (37.2%) | 54 (62.8%) |  |
| 1 | 4 | 0 (0.0%) | 4 (100%) |  |
| **Clinical stage**^4^ |  |  |  | < 0.001^3^ |
| I | 7 | 7 (100.0%) | 0 (0.0%) |  |
| II | 30 | 13 (43.3%) | 17 (56.7%) |  |
| III | 49 | 12 (24.5%) | 37 (75.5%) |  |
| IV | 4 | 0 (0.00%) | 4 (100%) |  |
| **Grade** |  |  |  | 0.678 |
| I/II | 24 | 8 (33.3%) | 16 (66.7%) |  |
| II-III | 18 | 8 (44.4%) | 10 (55.6%) |  |
| III | 48 | 16 (33.3%) | 32 (66.7%) |  |

^1^Median.

^2^The data of tumor size in three patients are not available.

^3^Statistically significant (*p* < 0.05).

^4^AJCC Cancer Stage Manual, 7^th^ Edition (2010).
