# Supplementary material for: SIRT1 suppresses the migration and invasion of gastric cancer by regulating ARHGAP5 expression
Source: Cell Death Dis. 2018 Sep 24;9(10):977. doi: 10.1038/s41419-018-1033-8 (PMC6155157; doi:10.1038/s41419-018-1033-8)
Supplement: Supplementary file 9 — Supplementary Table 3 [file 41419_2018_1033_MOESM9_ESM.docx]

**Supplementary Table 3. Univariate and multivariate analyses of overall survival of gastric cancer patients.**

| **Variables** | **n** | **Univariate Cox** | | | **Multivariate Cox** | | |
| --- | --- | --- | --- | --- | --- | --- | --- |
|  |  | **HR** | **95% CI** | ***p* value** | **HR** | **95% CI** | ***P* value** |
| **Gender** |  |  |  | 0.597 |  |  |  |
| Men | 70 | 1.000 |  |  |  |  |  |
| Women | 20 | 1.158 | 0.672-1.998 |  |  |  |  |
| **Age (year)** ^1^ |  |  |  | 0.078 |  |  |  |
| ≤ 66 | 45 | 1.000 |  |  |  |  |  |
| > 66 | 45 | 1.521 | 0.954-2.424 |  |  |  |  |
| **Tumor size (cm^3^)** ^1,2^ |  |  |  | 0.003^3^ |  |  | 0.096 |
| ≤ 32 | 45 | 1.000 |  |  | 1.000 |  |  |
| > 32 | 42 | 2.076 | 1.289-3.341 |  | 1.57 | 0.923-2.671 |  |
| **Tumor infiltration** |  |  |  | 0.004^3^ |  |  | 0.013^3^ |
| T1/T2 | 11 | 1.000 |  |  | 1.000 |  |  |
| T3 | 61 | 5.96 | 1.851-19.186 |  | 12.534 | 1.200-130.881 |  |
| T4 | 18 | 8.114 | 2.332-28.234 |  | 26.991 | 2.224-327.567 |  |
| **Local lymph node metastasis** |  |  |  | 0.002^3^ |  |  | 0.008^3^ |
| 0 | 23 | 1.000 |  |  | 1.000 |  |  |
| 1 | 16 | 1.68 | 0.764-3.692 |  | 4.628 | 1.724-12.427 |  |
| 2 | 25 | 2.548 | 1.282-5.065 |  | 14.586 | 2.745-77.498 |  |
| 3 | 26 | 3.622 | 1.824-7.191 |  | 15.031 | 2.782-81.213 |  |
| **Distant metastasis** |  |  |  | 0.120 |  |  |  |
| 0 | 86 | 1.000 |  |  |  |  |  |
| 1 | 4 | 2.251 | 0.809-6.261 |  |  |  |  |
| **Clinical stage**^4^ |  |  |  | 0.002^3^ |  |  | 0.278 |
| I | 7 | 1.000 |  |  | 1.000 |  |  |
| II | 30 | 3.162 | 0.740-13.504 |  | 0.228 | 0.012-4.380 |  |
| III | 49 | 6.804 | 1.638-28.269 |  | 0.056 | 0.001-2.176 |  |
| IV | 4 | 10.718 | 1.921-59.800 |  | 0.061 | 0.001-2.530 |  |
| **Grade** |  |  |  | 0.505 |  |  |  |
| I/II | 24 | 1.000 |  |  |  |  |  |
| II-III | 18 | 1.027 | 0.514-2.052 |  |  |  |  |
| III | 48 | 1.333 | 0.770-2.308 |  |  |  |  |
| **ARHGAP5 levels** |  |  |  | < 0.001^3^ |  |  | 0.001^3^ |
| Low | 32 | 1.000 |  |  | 1.000 |  |  |
| High | 58 | 4.946 | 2.729-8.961 |  | 3.14 | 1.617-6.095 |  |

n, Numbers of cases in each group.

HR, hazard ratio.

CI, confidence interval.

^1^Median.

^2^The data of tumor size in three patients are not available.

^3^Statistically significant (*p* < 0.05).

^4^AJCC Cancer Stage Manual, 7^th^ Edition (2010).
